# Forecasting of residential unit’s heat demands: a comparison of machine learning techniques in a real-world case study

**DOI:** 10.1007/s12667-023-00579-y

**Published:** 2023-05-09

**Authors:** Neele Kemper, Michael Heider, Dirk Pietruschka, Jörg Hähner

**Affiliations:** 1 Organic Computing Group, Universität Augsburg, Am Technologiezentrum 8, Augsburg, 86159 Germany; 2 Centre for Sustainable Energy Technology, Stuttgart University of Applied Sciences, Schellingstr. 24, Stuttgart, 70174 Germany

**Keywords:** Heat demand forecast, Machine learning, Time series, Smart buildings, Energy efficiency

## Abstract

A large proportion of the energy consumed by private households is used for space heating and domestic hot water. In the context of the energy transition, the predominant aim is to reduce this consumption. In addition to implementing better energy standards in new buildings and refurbishing old buildings, intelligent energy management concepts can also contribute by operating heat generators according to demand based on an expected heat requirement. This requires forecasting models for heat demand to be as accurate and reliable as possible. In this paper, we present a case study of a newly built medium-sized living quarter in central Europe made up of 66 residential units from which we gathered consumption data for almost two years. Based on this data, we investigate the possibility of forecasting heat demand using a variety of time series models and offline and online machine learning (ML) techniques in a standard data science approach. We chose to analyze different modeling techniques as they can be used in different settings, where time series models require no additional data, offline ML needs a lot of data gathered up front, and online ML could be deployed from day one. A special focus lies on peak demand and outlier forecasting, as well as investigations into seasonal expert models. We also highlight the computational expense and explainability characteristics of the used models. We compare the used methods with naive models as well as each other, finding that time series models, as well as online ML, do not yield promising results. Accordingly, we will deploy one of the offline ML models in our real-world energy management system in the near future.

## Introduction

The energy transition (the replacement of the use of fossil energy sources with an ecological, sustainable energy supply) is one of the most important environmental, economic, and sociological challenges this decade.

In addition to expanding renewable energies, increasing energy efficiency and reducing overall energy consumption are essential objectives. In particular, the building and private housing sectors have a high potential for energy savings. Therefore, the goal in the residential sector must be a reduction in heat and primary energy demand. In the future, buildings’ heating requirements must be covered entirely by solar, biomass, or geothermal energy. Accordingly, energy management concepts are being developed to ensure efficient and safe renewable energy use while fulfilling the thermal requirements of residents. However, these concepts necessitate methods for forecasting both the generation and the energy load [1]. Furthermore, they always presuppose individual boundary conditions, i.e., for private housing, the thermal comfort of the occupants must be ensured. So, developing accurate models to forecast the actual heat demand is essential.

For accurate heat demand prediction, the potential of data-driven methods has become apparent in recent years [2–4]. Unlike traditional engineering and physical methods, these techniques do not require detailed building data or extensive expertise to apply elaborate technical procedures, which is a significant advantage. Data-driven methods learn from real-time or historical data.

Using historical data, statistical models can be trained in a stationary learning environment. Batch learning techniques, known as (supervised) offline machine learning methods, and time series models are used to learn the best predictor from training data.

However, in many use cases, historical data is unavailable from the beginning of a system’s life. Moreover, the prediction of energy demand should be considered a non-stationary problem since unforeseen changes may occur over time, e.g., degradation of insulation, occupant changes, or general usage patterns. Generally, these issues are combined in the term concept drift [5]. To cope with these problems, models must be able to learn and evolve dynamically in an uncertain environment. In this work, the familiar issue of sensor drifts is, however, largely insignificant. The sensors capturing heat usage are calibrated for long-term use and regularly maintained or replaced after the guaranteed runtime. Whereas third-party weather data could theoretically suffer from sensor drifts, this is also unlikely to become a significant problem as those sensors are typically built for long-term stability.

A typical approach for handling (initially) low data availability and concept drift is the usage of online machine learning methods where the data is fed into the model sequentially whenever it becomes available (rather than at fixed timestamps—potentially even a single one before the first deployment—like in offline machine learning) to update its parameters and—hopefully—lead to the best possible predictor at each step.

In this work, we investigate the potential application of a large variety of different model producing methods in a newly built real-world residential setting, based on data we gathered from June 2020 to February 2022. These models should accurately forecast the heat demand of all units within the small complex. We make both the data gathered in this field study, as well as all of our results publicly available.

Regardless of the specific model type, this application domain—as it ensures the thermal comfort of occupants—requires its models to be well understandable for the engineers in the companies responsible. More significant issues could quickly terminate contracts and destroy business models, which in turn makes the application of more complex models less likely. Therefore, we discuss the employed models not only on their merits regarding predictive performance but also on their presumed transparency and explainability of decisions.

Moreover, heat supply is a critical task that must be ensured in any extreme or unusual situation (especially sub-zero temperatures). Therefore, we also examine how well the models predict heat consumption for data outliers.

In Sect. 2, we reintroduce the task of heat consumption forecasting. Section 3 gives a more detailed overview of the aims of this specific work with Sect. 5 introducing the data set we first gathered and then investigated the forecasting methods on. This field’s state-of-the-art and other recent approaches are summarized in Sect. 4. Section 6 gives an overview of the employed methods and their potential merits, whereas Sect. 7 introduces our experimental approach at evaluating these methods in relation to our field study’s data. The results are first presented in Sect. 8 and then discussed in detail (especially regarding the predictive errors, the usage in embedded systems, and the explainability of models) in Sect. 9. In our data, we found some outliers that were quite hard to predict correctly. These and the models’ results are discussed in Sect. 10. Section 11 concludes this paper and gives an overview of our results and an outlook on the next steps within this field study.

## Problem description

Most data-driven models require large and diverse sets of training data for accurate heat consumption prediction. These datasets often contain data from several years to represent seasonal patterns and trends. However, for more specialized use cases, such as individual neighborhoods, these data sets are mostly unavailable. Usually, when new energy systems are commissioned, no training data has been gathered, even if energy management systems had been in place before. Even during the active operation of modern systems, the data sets grow slowly. Additionally, the data can not initially reflect any long-term seasonality or trends. The forecast quality drops significantly when an unfamiliar situation occurs, e.g., the first summer/winter or some concept drift.

The heat consumption can be described by a continuous function that models the relationship between the heat consumption $y \in {\mathbb {R}}$ and a set of *k* variables $x \in {\mathbb {R}}^{k}$ for a time *t*:

1 $$\begin{aligned} f(x_t) \rightarrow y_t \end{aligned}$$

The heat consumption is modeled as a sequence of data points over time for time series analysis models. The time series is decomposed into the deterministic trend ${m_t}$, seasonal components ${s_t}$ and a random, stationary component (error) ${\epsilon _t}$.

2 $$\begin{aligned} y_t = m_t + s_t + \epsilon _t \end{aligned}$$

## Aim of research

This research aims to evaluate models predicting heat requirements for an energy management system, which regulates a central heating system and distributes heat to multiple residential units. The models have to achieve good predictive performance despite limited training data (cf. Sect. 5), a situation typically encountered in new energy management systems or newly built or renovated units. If a new and unknown situation occurs, the investigated models must generalize well, i.e., make stable predictions with a small error value. Based on the models’ forecasts, a schedule spanning the next 48 to 72 h is created for the energy management systems. Subsequently, the schedule gets updated every 24 h as more up-to-date (weather) data is available. In line with our field study’s requirements, the duration of the schedules is chosen to ensure that the power systems can continue to run automatically in the event of internet failures. The field of application is the load and storage management, respectively, energy management systems, for buildings and quarters. Specifically, the results of this research will later be used in the area of a cloud application with distributed edge devices. It is analyzed whether the model computations can be performed directly on the embedded systems, which often have low computational and memory performance, or should be outsourced to an external server. In addition, the models are examined to determine whether the model’s predictions can be explained and understood by (non-specialist) persons responsible for the systems and the heat supply.

## Related work

In recent years, many studies on energy load forecasting have been published. The data-driven approaches can be classified into statistical and machine learning (ML)-based methods.

Time series models are widely used in statistical methods. For these methods, the consumption is modeled as a time series. In general, the forecast horizon for load forecasts for heat (or electricity) is divided into short-term and long-term, where short-term forecasts give a minutely or hourly forecast in a horizon is up to 24 h, whereas long-term predictions refer to load forecasts for, typically, 1 week but also up to one or more years. The goal of short-term horizons is to optimize the day-to-day operation of energy systems while long-term model can be used for the planning of energy systems. In this study, we aim at making short-term predictions, although, the following models after frequently used in both settings. Particularly frequently studied time series models are ARIMA [6–9] and its improvement SARIMA [10], and Exponential Smoothing [11–14]. The development of the BATS models (Box-Cox transformation, ARMA residuals, trend, and seasonality) and the TBATS models (trigonometric seasonal BATS) was a significant advance in the field of time series forecasting techniques. BATS and TBATS can be used to model time series with multiple complex seasonalities [15, 16]. The TBATS model has excellent forecast accuracy and offers a possibility for long-term load forecasting [17–19]. An alternative, based on grey system theory and Markov chains rather than ARMA, are Grey-Markov models (GM) [20–22]. Studies that have compared GM models with ARIMA models have concluded that the predictive performance of both models is comparable, with ARIMA being slightly better than GM [23] or vice versa [24]. However, GM’s predictions often undershoot, which is detrimental to supplying sufficient heat, while ARIMA tends to overshoot [24]. Also, GM is somewhat more computationally intensive [24]. Another basic statistical analysis method is to model the load forecasts linearly, as with Linear Regression (LR) [25, 26], Recursive Least Squares (RLS) [27–29], fuzzy LR methods [30] or Polynomial Regression [31].

A wide range of different approaches to ML has been pursued. Often evaluated ML models are Support Vector Regression (SVR) [32–34], respectively Support Vector Machines [35–37], Random Forest Regression (RFR) [38, 39], or Kernel Ridge Regression (KRR) [40]. The comprehensive literature review on energy demand forecasting by Ghalehkhondabi et al. [41] shows that artificial neural network (ANN) models perform very well in this domain. This conclusion is also confirmed by later studies that have investigated ANNs [42–45], Long Short-Term Memory (LSTM) networks [34, 46–48] or Convolutional Neural Networks (CNNs) [49, 50].

Different ML methods are combined to improve the prediction quality of ML models to reduce their respective drawbacks. In short-term load forecasting, SVR is often combined with other ML methods [51–53]. Another option is to combine LSTMs with CNNs [54–57]. The conventional LSTM neural network is extended by a preprocessing phase using a CNN.

Beyond the already presented methods, authors recently took a variety of different approaches towards predicting energy demand: [58] combines ANNs with metaheuristic algorithms, including artificial bee colony optimization, particle swarm optimization, an imperialist competitive algorithm, and a genetic algorithm. In [59], further development of CNNs for limited data is presented. Kannari et al. [60] combine physics-based modeling and ML to forecast the energy consumption of buildings. Potočnik et al. [61] investigate a multi-stage ML-based approach for short-term heat demand forecasting. The approach includes feature extraction and different ML models for forecasting. A similar approach is taken by Golmohamadi [62]. In [63] and [64], probabilistic approaches are presented and combined with ML models. Recently, studies have been published that take a similar approach to our present study. Kurek et al. [65] investigate various regression models, deep neural networks, and models using fuzzy logic for heat demand forecasting for the Warsaw district heating network, which supplies heat for domestic and heating purposes. They divide the year into summer, winter, and intermediate seasons and evaluate the models for each season for a 72-h horizon.

## Case study and data set

**Fig. 1:**
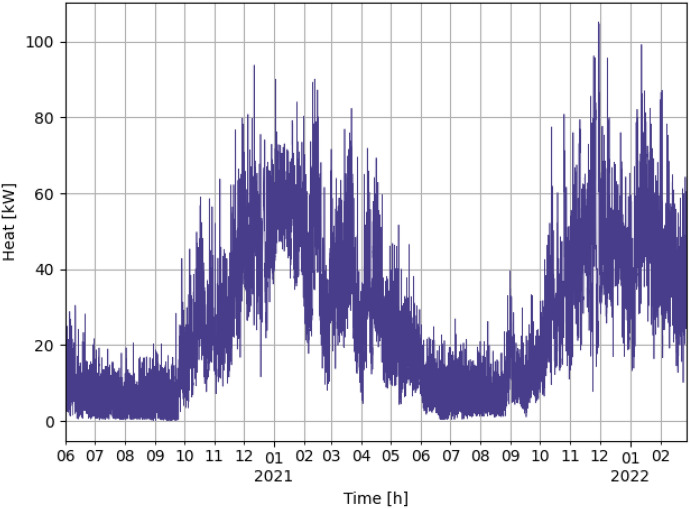
The scaled hourly heat consumption measured in kilowatts [kW], from 1. June 2020, to 28. February 2022

The data was gathered in a newly built residential quarter (completion 2019) in southern Germany near Munich. The quarter contains 66 residential units, and the heat meter is located behind a heat accumulator and records the domestic hot water supply and the space heating demand. A controller keeps the flow after the heat accumulator at a constant temperature of 48$^\circ$C. While in the original data, the heat demand of all 66 apartments was recorded individually for billing reasons, we want to stress that in this study we aim at predicting the load at the central heating system. The controller of said system distributes heat to the apartments individually but only the overall required heat is relevant for planning purposes. From a data science perspective, this also has the advantage that we can better compensate for any erroneous data or data failures from individual apartments, therefore also adjusting for noise. The heat demand is examined in a 60-minute interval and measured in watt-hours [*Wh*]. Thus, it is not the behavior of the residents that is predicted, but the required output of the heating system in 1 h. The scaled heat consumption is shown in Fig. 1. The hydraulic diagram of individual housing stations where a heat meter is installed can be found in the supplementary information’s^1^ Sect. 1.

The data was collected in a period from 01. June 2020 to 28. February 2022. The limits of the period are set by the commissioning of the monitoring system and the first draft of this paper. In total, 15,228 data points were collected during this period.

Eight parameters are available as input for the models: Month of the year, day of the week,^2^ the hour of the day, outside temperature, solar radiation, heat consumption 24 h ago, average heat consumption over the last 24 h, and the degree hour. The degree hour is the difference between the target inside and the measured outside temperatures. It is only calculated for days whose average outside temperature is below a heating limit. A target inside temperature of 21$^\circ$C and a heating limit of 15$^\circ$C is assumed. Outside temperature and solar radiation were retrieved from the weather API Weatherbit.^3^ Sect. 2 of the supplementary information contains Fig. 1’s corresponding parameters temperature, solar radiation, heat consumption 24 h ago, average heat consumption over the last 24 h, and degree hour.

## Forecasting concepts

In this section, we introduce the various forecasting concepts from traditional statistics and modern machine learning (ML) that were used in this work. While none of them are new, this section improves the self-containedness of the paper and hopefully allows readers from a civil engineering background to better understand and follow this work and maybe reapply it to their own data.

In general, forecasting techniques can be divided into two main types: simple statistical or parametric models, and ML-based models.

Classical models, such as (S)ARIMA, Exponential Smoothing, and LR, use historical data for mathematical combinations to forecast heat consumption. Their advantage is that the estimations of the parameters are easily interpretable.

However, as the complexity of the forecast data increases, these methods become less reliable. A transition from linear to nonlinear models is necessary. ML-based methods are generally adaptive and robust to noisy data due to their ability to generalize from observed patterns. Often used ML methods in load forecasting are ANNs, SVR and RFR. Within the family of ANNs, different architectures are common: The traditional fully connected network, as well as CNNs, LSTMs, or combinations of these.

If the training data $D=\{(x_1,y_1),...,(x_t,y_t)\}$ is available in sequential order, a model to predict the next time step $t+1$ can be learned via online learning. Online learning updates the predictor in real-time, always incorporating the newest available data, which can protect against the influences of data drifts but might struggle with strong seasonalities in the data.

In our work, we incorporate a variety of online learners: RLS, Stochastic Gradient Descent trained linear models (SGD), and three types of ANNs. As fully connected ANNs have some limitations in an online learning environment, two additional concepts are investigated: an ANN combined with an Experience Replay (ER)–like buffer and the online framework based on Hedge Backpropagation (HBP) presented by [66].

The following is a brief outline of the methods examined. We sort them from time series over offline ML to online ML, and within their respective category by model complexity:

***Holt-winter smoothing***

Exponential Smoothing is a powerful time series forecasting method for univariate data that is often used as an alternative to the autoregressive approach. Exponential Smoothing combines the advantages of flexibility, reliability of predictions, and low cost. Holt-Winter Smoothing (HWS) [67, 68] is an extension of simple exponential smoothing for trends and seasonal patterns.

***Seasonal autoregressive integrated moving average***

Autoregressive Integrated Moving Average (ARIMA) is a statistical model for non-stationary time series. Seasonal ARIMA (SARIMA) takes the non-seasonal components of the ARIMA and adds a seasonal term. The seasonal term is very similar to the non-seasonal components of the model, but it includes a backward shift by a seasonal period [69].

ARIMA and exponential smoothing use complementary approaches to predict time series. Exponential smoothing models describe the trend and seasonality in the data, while ARIMA models describe the autocorrelation in the data [69].

***Linear regression***

Linear Regression (LR) is the simplest approach to model the relationship between a set of independent variables ${x^T = (x_1, x_2,..,x_p})$ and a dependent variable *y*. This is in contrast to the aforementioned time series approaches, where predictions on *y* were made based on previous values for *y*, assuming that there is a sequential order. Fitting a linear model to a given data set requires the estimation of regression coefficients, typically by minimizing the squared error terms.

***Kernel ridge regression***

When a learning task can not be modeled using a linear function, a kernel can be used to transform the data into a higher dimensional space, called kernel space, where the data can be modeled linearly. A fundamental algorithm to be kernelized is Ridge Regression, which attempts to solve the frequent problems of multicollinearity and high variance in LR by including shrinkage methods and L2 regularization in the updates. Kernel Ridge Regression (KRR) combines Ridge Regression with a kernel.

***Support vector regression***

Support Vector Regression (SVR) is a generalization of traditional support vector machines for classification and supports both linear and nonlinear regressions. SVR formulates the function approximation itself as a linear function, where the data is mapped into kernel space for a nonlinear function to achieve lower errors.

***Random forest regression***

In Random Forest Regression (RFR), a collection of uncorrelated decision trees is built and their individual predictions are averaged to produce an accurate prediction of the dependent variable *y*.

***Deep neural networks***

A fully connected layer is a way to arrange neurons in an ANN where all neurons of a layer are connected to all neurons of the next layer (with individual weights). A sufficiently large network (having multiple fully connected layers in sequence) is commonly referred to as a deep neural network (DNN). Although this term nowadays often refers to networks with a wide variety of layers (as long as they are numerous), we stick to the original sense of multiple fully connected layers for this work.

***Convolutional neural networks***

Convolutional Neural Networks (CNNs) comprise three types of layers: convolutional Layers, pooling Layers, and fully connected layers. Parameters of the convolutional layer focus on the use of filter operations. A discrete convolution calculates the activation of the neurons, instead of matrix multiplication as in other ANNs. A pooling layer gradually reduces the dimensionality of the representation, thus reducing the number of parameters and computational complexity of the model.

***Long short-term memory***

ANNs have limitations in solving sequential data problems, such as time series. Long Short-Term Memory (LSTM) is a recurrent network using a chain-like structure of repeating cells. These cells store important information from previous training steps, enabling the learning of long-term dependencies, and use a feedback loop to accept a sequence of inputs.

***Recursive least squares***

Recursive Least Squares (RLS) is an extension of the Least Squares method, with a recursive algorithm to design an adaptive filter where the parameters are updated iteratively, enabling this method to optimize a linear function in an online learning setting.

***Stochastic gradient descent***

Gradient descent is an iterative optimization algorithm for differentiable functions. The function is minimized by updating the parameters proportionally to the negative of the gradient of the target function. Stochastic Gradient Descent selects a random subsample of the data to compute the gradient. When used in an online ML setting, the most recent data point is used. In this study, we use it to optimize a *linear function* to which we refer by SGD to make it easier distinguishable from the offline ML linear Regression model.

***Online deep learning***

The straightforward approach to transferring the training process of a fully connected network to an online learning environment is to apply the backpropagation algorithm to a single instance rather than batches or mini-batches of data. We refer to this as Online Deep Learning (ODL).

***Experience replay***

Experience Replay (ER) is a technique designed to resolve the problem of catastrophic forgetting in ANNs, where subsequent updates of the model lead to a forgetting of previously learned information. This problem is especially big when using very small online updates rather than shuffled mini-batches and can lead to the loss of seasonal information. In this paper, we employ an ER-like technique to a DNN (ODL-ER), where we add new training data to a fixed length FIFO buffer from which we randomly sample data points and merge them with the newest data points into a mini-batch to perform model updates using backpropagation.

***Hedge backpropagation***

ODL, as described above, has some critical limitations. The main challenge is to choose the network’s ideal architecture (complexity) in advance of the training. If the model is too complex, the learning process converges too slow, breaking an essential requirement property of online learning. However, if the model is too simple, complex patterns cannot be learned. In traditional model selection, this problem would be solved using validation data, which is unavailable in an online environment. Sahoo et al. [66] present a framework that attempts to solve these challenges by developing an ANN that is adaptive in complexity. The framework was initially developed for classification problems but is applied to the continuous regression problem of heat load forecasting in this work.

The existing ANN architecture is adapted by connecting each hidden layer to an output layer. Instead of the standard backpropagation, Hedge Backpropagation (HBP) is used, which evaluates the performance of the output layers in each online round and extends the backpropagation to train the ANN online. The outputs of the different depths are optimally utilized by the hedge algorithm [70]. This allows training an ANN with adaptive capacities to simultaneously share knowledge between shallow and deep networks. The architecture of multiple depths makes the learning process robust to vanishing gradients and decreasing feature reuse.

## Methodology

The basic design of experiments is the same for all methods examined and outlined below. However, the evaluation of prediction quality distinguishes between time series models, offline ML methods, and online ML methods. On the one hand, from an algorithmic perspective, the heat demand is modeled according to the different approaches of taking data between the models. More importantly, the application options differ between the three approaches. Time series models do not require additional features beyond the raw consumption, whereas offline and online ML methods use additional features (as outlined in Sect. 5). If such data is available both approaches are theoretically viable. However, offline models would be employed in settings with low concept or sensor drifts for which plenty of historical data is available, whereas online models would be used when strong drifts occur or data has not (yet) been gathered sufficiently.

An overview of the main software libraries used can be found in the supplementary information’s Sect. 3.

### Experimental design

The data set is divided into summer months and winter months, corresponding to not-heating season and heating season, respectively. The allocation was made based on the specific measured heating demand. The winter months are defined from 21. September to 04. May. Accordingly, the summer months are set to 05. May to 20. September. We want to stress that this means that we have different lengths of application of a supposedly seasonal model, as well as differing amounts of data. Importantly, it also emphasizes the higher importance of lower prediction errors in what we refer to as winter/heating season.

We use the entire data set to train and validate the models (without separate holdout sets). Models trained on both summer and winter data are referred to as *all* in the following. In addition, we examine how the models trained on the entire data perform when being evaluated on the summer months (*all-summer*) and the winter months (*all-winter*). Furthermore, other models are trained and assessed using only data from summer (*summer*) or winter months (*winter*). This gives an idea whether it would be beneficial to train separate models for the different seasons.

***Time Series Models*** These are trained with the first-year data (June 2020 to May 2021) and evaluated on the second-year data (June 2021 to February 2022), with the usual distinction of summer and winter months.

The optimization of hyperparameters of the time series models is performed using a grid search approach. The performance of the grid search algorithm for the different hyperparameter settings is measured using the second-year data. Preselection of the SARIMA parametrization is performed using the Box-Jenkins method.

***Offline ML methods***

The data is randomly divided into training and test data with a ratio of 4 to 1. The data partitioning uses 30 ascending seeds, resulting in 30 different training and testing datasets. This process is also known as monte-carlo cross-validation. The different methods are each trained on each of the 30 training datasets and evaluated with the corresponding test dataset. The mean performance is then calculated and reported over those runs.

The optimization of hyperparameters of the models was performed with the framework KerasTuner [71] using Bayesian Optimization.

***Online ML methods***

The data is presented in sequential order to the algorithms, simulating an in-vivo deployment. A model is trained with the data of the first 24 h. Next, the forecast quality is evaluated with the data for the next 24 h. The model is then updated with the next 24 data points. This updating, forecasting, and evaluation cycle continues until no new data is available. For all methods, this experimental run is repeated 30 times and a mean performance is calculated and reported.

To optimize the hyperparameters of ODL and HBP, KerasTuner with Bayesian Optimization is used. For the ANN of ODL-ER, we adopt the parameterization of ODL. For the parametrization of the ER component, we use grid search, which is also used for RLS and SGD. We use Grid Search for RLS, SGD, and ER because they cannot be straightforwardly optimized using KerasTuner and, due to a comparatively small hyperparameter space that needs to be examined, the easier-to-use and follow Grid Search can be used comfortably while avoiding performance problems.

### Metrics

The prediction performance of the models is examined using various metrics.

The *Root Mean Squared Error (RMSE)* is used to measure the prediction quality.

3 $$\begin{aligned} RMSE = \sqrt{\frac{1}{n}\sum ^n_{i=1}({\hat{y}}_i - y_i)^2} \end{aligned}$$

The *Mean Absolute Scaled Error (MASE)*, unlike the RSME, is a scale-independent measurement. The MASE is the mean absolute error of the predicted value divided by the mean absolute error of a naive benchmark model. We employ the seasonal MASE where the naive model prediction is created at each time step by equating the current prediction with the corresponding value of the last time step. We report this additional metric as the scaling indicates how well the model compares to a simple model and, therefore, determine if it is worthwhile to even consider the model for usage.

4 $$\begin{aligned} MASE = \text {mean}\left(\frac{\left| e_j\right| }{\frac{1}{T-m}\sum ^T_{t=m+1}\left| y_t - y_{t-m}\right| }\right) \end{aligned}$$

The numerator $e_j$ is the absolute prediction error for a given period (with *J*, the number of predictions), defined as the actual value ($y_j$) minus the predicted value (${\hat{y}}_j$) for that period: $e_j = y_j - {\hat{y}}_j$. The denominator is the mean absolute error of the one-step “naive forecast method" on the training set, which uses the actual value of the previous season as the forecast: ${\hat{y}}_t = y_{t-m}$, where *m* is the seasonal period. As a seasonal period, we choose $m=1$, that is, the last known value, for both the time series and the online models. For offline ML models the MASE typically uses the mean of the data as a naive model. Thus, we use it as well in this work.

For the offline and online ML methods, the scattering measure of the *Mean Absolute Deviation from the Median (MAD)* over the predictions of the different runs is calculated.

5 $$\begin{aligned} MAD = \text {mean}\left(\left| y_{i,j}-{\tilde{y}}_j\right| \right), \end{aligned}$$

where $y_{i,j}$ is the prediction of run *i* at time index *j* and ${\tilde{y}}_j$ is the median of the predictions of all runs for time index *j*, whereas the mean averages over all time indices.

To better compare the forecasting performance of different models, it is tested whether the different results between two models are statistically significant or due to random chance. This is tested using a statistical significance test. For normally distributed competence values, the *Student’s T-Test* can be chosen; otherwise, the *Mann–Whitney U-Test* is appropriate. The selected significance level of 0.05 is adjusted with the Bonferroni correction.

An overview of the search spaces for the parametrization and the optimal found parametrization of the investigated models can be found in supplementary information’s Sect. 4.

## Results

In this section, we present the results of the experiments for the different methods, grouped by the three families of algorithms, producing different scenarios of a deployed prediction system (cf. Sect. 7).

In preliminary investigations, we could show that the quality of 24-h and 48-h forecasts hardly differ. Therefore, the 24-h forecast, which is the—at the moment—typical application in a real-world scenario, is presented here in more detail. A detailed comparison of the metrics for a 24-h and 48-h prediction for the offline and online ML models can be found in the supplementary information’s Sect. 5. In the future, the 48-h forecast might become more important. Therefore, we want to stress that the following findings do transfer.

For all models examined, the RMSE value is lower in the summer months than in the winter months, as can be seen in Tables 1, 3, and 6. This is in no way surprising, as heat consumption is lower in the summer months than in winter (cf. Fig. 1, as there is no need for space heating. However, the relative deviation of the forecast from the actual consumption is higher in summer.

The time series models, the LSTM, and the SGD have the highest RMSE values among all the examined models and do not seem to be competitively performing options (even within their respective scenarios) (Table 2).

### Time series

**Table 1:** Comparison of the RMSE values in kWh of the two time series methods for the different periods summer, winter, and all year. The best-performing model’s value for the respective period is highlighted in bold

Time Series - RMSE [kWh]		
	HWS	SARIMA
Summer	8.13	**6**.**93**
Winter	21.01	**15**.**68**
All	21.84	**15**.**72**
All-summer	**10**.**95**	14.64
All-winter	26.96	**16**.**44**

**Table 2:** Comparison of the MASE values in kWh of the two time series methods

Time series - MASE [kWh]		
	HWS	SARIMA
Summer	2.02	**1**.**65**
Winter	3.02	**2**.**24**
All	3.66	**2**.**79**
All-summer	**3**.**25**	4.53
All-winter	4.01	**2**.**28**

The time series models are utterly unconvincing. While SARIMA mostly outperforms HWS, the errors Table 1 are still very high. MASE values indicate that the models perform worse on average than the naive model, which always returns the previous value. While the seasonal experts outperform the general model, neither is application ready.

### Offline machine learning

**Table 3:** Comparison of the RMSE values in kWh of the seven offline ML methods

Offline Machine Learning - RMSE [kWh]							
	LR	KRR	SVR	RFR	DNN	CNN	LSTM
Summer	4.05	**3**.**53**	3.71	3.58	3.9	3.78	5.56
Winter	9.33	**7**.**33**	8.12	7.58	9.11	8.34	15.91
All	7.82	**6**.**11**	6.76	6.37	7.79	7.2	17.61
All-summer	4.39	**3**.**54**	3.67	3.61	4.4	3.94	15.42
All-winter	9.39	**7**.**3**	8.14	7.63	9.33	8.67	18.9

Overall, offline ML models, except for LSTM, perform slightly better than their online counterparts (cf. Tables 3 and 6, respectively), although we want to stress the caveat that those can not be deployed together with the energy management system but require longer periods of data gathering beforehand. LSTM performs comparable to the time series models. The—on average—best-performing model is KRR, a relatively simple and comparably well-interpretable model. RFR, a substantially more complex model, performs similarly well.

**Table 4:** Comparison of the MAD values in kWh of the seven offline ML methods

Offline machine learning - MAD [kWh]							
	LR	KRR	SVR	RFR	DNN	CNN	LSTM
Summer	**0**.**05**	0.26	0.3	0.18	0.17	0.25	0.05
Winter	**0**.**09**	0.63	0.56	0.35	0.28	0.91	0.25
All	**0**.**06**	0.49	0.35	0.31	0.2	0.64	0.09
All-summer	**0**.**05**	0.29	0.2	0.17	0.15	0.47	0.1
All-winter	**0**.**07**	0.62	0.45	0.4	0.23	0.74	0.09

However, accounting for the MAD values (cf. Table 4), where RFR is indicated at more reliably arriving at those scores (compared on different sets of randomly split data), might motivate the usage of RFR over KRR. Interestingly, both do not perform substantially better than a simple LR which also exhibits the most reliable performance (indicated by the very low MAD values (0.05 kWh - 0.09 kWh)). CNNs also show a similar—albeit slightly worse—performance but are quite susceptible to different splits. Performance trends between the five settings are similar and there does not seem to be a benefit of creating specialized models for summer and winter over just training one generalist on all data that then evaluates on whatever the current season requires.

**Table 5:** Comparison of the MASE values in kWh of the seven offline ML methods

Offline Machine Learning - MASE [kWh]							
	LR	KRR	SVR	RFR	DNN	CNN	LSTM
Summer	0.66	**0**.**56**	0.59	0.57	0.63	0.61	0.9
Winter	0.52	**0**.**4**	0.44	0.41	0.5	0.45	0.95
All	0.34	**0**.**26**	0.28	0.27	0.33	0.31	0.89
All-summer	0.72	**0**.**57**	0.58	**0**.**57**	0.73	0.64	0.99
All-winter	0.52	**0**.**4**	0.44	0.41	0.51	0.47	0.95

In contrast to our findings above on time series, MASE values (cf. Table 5) indicate that using relatively complex machine learning with additional input information proves beneficial over using the mean as the base forecast.

To reiterate, this result illustrates that some meaningful pattern could be extracted from the (additional) data.

### Online machine learning

The online ML models display (cf. Table 6) slightly higher RMSE values than offline ones.

**Table 6:** Comparison of the RMSE values in kWh of the five online ML methods

Online machine learning - RMSE [kWh]					
	RLS	SGD	ODL	ODL-ER	HBP
Summer	4.16	5.13	4.42	**3**.**83**	4.29
Winter	9.58	25.01	10.6	**8**.**64**	10.59
All	7.49	15.55	8.5	**6**.**61**	8.13
All-summer	**4**.**15**	5.07	4.37	**4**.**15**	4.29
All-winter	9.57	22.09	10.46	** 8.68**	10.53

However, the SGD model diverges strongly and performs similarly to the time series models. For the summer months, the RMSE values of all other online models are in a comparable range and only marginally differ from those of the offline ML models. For the winter months, however, this difference increases noticeably. Interestingly, as with offline ML models, seasonal experts do not massively outperform their generalizing counterparts (Tables 7, 8). Overall, the best-performing model seems to be ODL-ER, although with a slightly wider distribution over the different runs.

**Table 7:** Comparison of the MAD values in kWh of the five online ML methods

Online machine learning - MAD [kWh]					
	RLS	SGD	ODL	ODL-ER	HBP
Summer	**0**	1.6	0.09	0.1	0.05
Winter	**0**	18.59	0.19	0.31	0.2
All	**0**	10.06	0.15	0.2	0.12
All-summer	**0**	1.44	0.08	0.15	0.1
All-winter	**0**	15.44	0.19	0.23	0.13

As RLS does not involve a stochastic component, its MAD of 0 is unsurprising. None of the online ML methods beat the naive model, the actual value from 1 h ago, which is somewhat discouraging their usage.

**Table 8:** Comparison of the MASE values in kWh of the five online ML methods

Online machine learning - MASE [kWh]					
	RLS	SGD	ODL	ODL-ER	HBP
Summer	**1**.**04**	1.24	1.08	1.16	1.07
Winter	1.44	4.15	1.58	**1**.**24**	1.6
All	1.29	2.7	1.37	**1**.**23**	1.39
All-summer	**1**.**04**	1.21	1.06	1.22	1.06
All-winter	1.44	3.63	1.56	**1**.**23**	1.59

## Discussion

This section discusses the experimental results and the advantages and disadvantages of the methods for the specific real-world use case.

In our use case, as well as many similar ones, the most commonly used controller is the single-board computer PhyBOARD-Regor from Phytec Messtechnik GmbH.^4^ Here, a phyCORE-AM335x is used as the processor and 512 MB NAND Flash and 512 MB DDR RAM are integrated as memory modules. The controllers have limited computational and memory power, which is a critical limitation for the direct use of the models in embedded systems. Keep in mind that besides doing statistical inference using the trained models (and maybe even model training), these controllers also need to do their original task allocating substantial shares of their available resources.

A critical constraint is the prediction of peak demand. Accurate forecasting of peaks is essential for safe and reliable scheduling of heat supply at peak times to ensure the thermal needs of residents are met.

For all methods and models, the deviation of the prediction for the summer months is higher than the deviation in the winter months. This is particularly well shown by the MASE values of the offline ML methods for the summer and winter months. MASE values are higher in the summer months with an average of 0.67 than in the winter months with 0.53.

The prediction errors are lower in the summer months than in the winter months, but if the low consumption in the summer months is taken into account, the prediction is inaccurate. This is because the heat demand in the summer months consists mainly of hot water demand and the drawing patterns vary considerably. Due to the low number of apartments and residents, water consumption deviating from the norm strongly influences the summer months’ heat demand. The domestic hot water demand is difficult to predict because single deviations strongly influence existing patterns. While hot water shows inconsistent trends all year, summer vacations additionally disrupt general everyday patterns for occupants. A high variation of the prediction for the summer months cannot be avoided in this use case. The additional space heating demand—related to the outside temperature—in the winter months compensates for those irregularities.

### Time series

The advantage of both time series models is that they are easy to understand, apply, and implement. However, time series analysis techniques require large amounts of error-free data that depicts long-term trends and patterns, which are unavailable in this use case. As these are mathematically simple models, they fail to model more complex trends and patterns, as illustrated by the high RMSE values of the two methods. Therefore, both models are ill-suited for the use case examined.

The advantages and disadvantages of the two time series methods evaluated are briefly discussed in detail:

#### Holt–Winter Smoothing

HWS emphasizes recent observations. The predictions lag behind the actual trend as a side effect of the smoothing process, which also neglects highs and lows caused by random fluctuations. This is also reflected in the high error value. HWS has many critical limitations for practical application. The lagged trend prevents short-term but essential changes in the trend, such as a sudden cold snap, from being incorporated into the prediction. The neglect of lows and highs is particularly critical for forecasting peak demand.

#### SARIMA

SARIMA makes stable estimates of the trend and seasonal patterns. However, it can only extract linear relationships within the time series. For more complex patterns and trends in heat consumption, SARIMA reaches its limits, as seen in high error values. The coefficients are difficult to interpret, and there is a risk that parameters are incorrectly fitted.

As noted in the results, the seasonal expert models of time series methods perform significantly better than their general counterparts. This is likely due to the limited training data. The general model cannot learn the global seasonal pattern of heating and non-heating seasons. The high error values particularly show this for the summer months in the general model. The models were last fitted with data from the winter months. For the following summer months, the models automatically assume a heating period. If only limited data is available, separate models should be learned accordingly. Whether, after a few years, a general model could perform on par with the offline ML approaches remains unknown. This might be interesting as to the low computing power required, the time series models can be trained and applied directly to the controller.

However, regardless of this, none of the time series models was able to outperform the naive model (or even be competitive). Therefore, their application should not be further considered.

### Offline machine learning

ML models easily recognize trends and patterns in data. They are good at learning connections and relations in multi-dimensional and multivariate data and can model complex consumption patterns.

However, offline ML models face some limitations to our specific use case. To achieve a good prediction quality, the methods require detailed data, which is not always available in a real-world scenario. With the availability of new data, the models have to be re-trained and often re-parametrized, which involves additional computational effort and data science expertise. In addition, the models lag behind the latest observations and thus cannot respond quickly to concept drifts.

In the following, the offline ML methods investigated are discussed in more detail.

#### Linear regression

LR performs well when the data set is linearly separable. The assumption of linearity is also the major limitation of LR because a linear relationship between the variables in real heat consumption is rarely given. This explains the high error values of LR in the winter months. The heat demand can be modeled linearly to a certain extent for the summer months. The prediction error of the LR for the summer months is in the same order of magnitude as that of all other ML models, except the LSTM models. The LR is particularly sensitive to noise, overfitting, outliers, and multicollinearity.

Summarizing, LR is easy to implement, interpret, and efficient to train, while it can be used directly on controllers and is easy to understand even for non-specialized people. However, it does not sufficiently capture the important patterns to model winter month heat consumption.

#### Kernel ridge regression

KRR prevents overfitting to some extent by L2 regularization on its updates. It can solve non-linear problems, resulting in lower error values for the winter months. The calculation of KRR is efficient on a low-dimensional data set such as this one. With a large amount of training data, the memory requirements and computing power are high. The memory required for the kernel matrix grows quadratically, and the computational power needed for the model grows cubically for the model to the size of the training sample [72].

#### Support vector regression

SVR can also solve non-linear problems and is somewhat robust to outliers, shown in average RMSE values for the winter months. The SVR benefits from its simple implementation. Compared to other regression techniques, it performs few computations and is more computationally efficient than KRR and RFR, making it more suitable for direct application on embedded systems. High accuracy requires a lot of memory for the support vectors and is, therefore, unsuitable for embedded training of larger data sets.

#### Random forest regression

RFR can solve non-linear problems efficiently by combining the outputs of multiple decision trees, reducing overfitting and variance. The low RMSE and MAD values confirm this for RFR in the results. Also, RFR can automatically handle missing data and is robust to outliers, as shown by the small error values for the winter months. It is also little affected by noise and is very stable, even with new data. This is an essential aspect of practical applications, where clean data is not guaranteed and stable prediction in unknown situations is necessary. However, RFR is quite complex to interpret and, while this is theoretically doable, it is usually not realistically achievable for non-data scientists. Additionally, training time is often long, requiring moderately high compute, making it unsuitable for training on the controllers when a large training data set is provided.

#### Deep neural networks

Sufficiently large DNNs have a robust non-linear mapping capability and a high tolerance to complexity in the data. DNNs can learn prediction models well, even if the data does not have constant variance or noise terms are unavailable. Based on these characteristics, a DNN should, in theory, be ideal for forecasting heat consumption. However, this is not confirmed by the below-average results. Typically, DNNs not only require more data than other ML methods, they are also heavily influenced by bias in the data, leading to overfitting and poor generalization. This limitation, together with the unsolved problem of explaining a DNN’s predictions, negates the advantages of a DNN for the examined use case. Likely, the data set examined in this study is too small for a good predictive function to be learned, as shown by the high error values.

CNN and LSTM inherit the advantages and disadvantages of the simpler DNN. CNN has a more complex architecture, making parametrization even more critical. LSTM is already even more prone to overfitting because typical regularization techniques, such as dropout layers, are challenging. This is a probable reason for the poor performance of LSTM in this study. LSTM is hardware inefficient and cannot be trained and, for more complex models, maybe not even be deployed on the embedded hardware.

In addition, NNs are very time-consuming to build and require high computing power. High computing power is especially critical because, in practice, as in this case, embedded systems with low computing capacity are used. However, this is not yet a debilitating problem for the use case considered in this paper, as the models could be trained remotely and then deployed, as for most models, inference is substantially cheaper and should be doable on most commonly-used systems.

#### Statistical analysis

Frequentist statistical significance tests show that the RMSE values of almost all models differ, regardless of their complexity and different approaches. The null hypothesis is not rejected between LR and DNN trained and evaluated on the entire dataset. Also, the null hypothesis is not rejected for the KRR and RFR, as well as for the CNN and LR trained and evaluated in the summer months. It cannot be said with certainty that these models have a better prediction quality. However, as those tests can offer misleading results, we visually investigate the distributions of the results in the following.

**Fig. 2:**
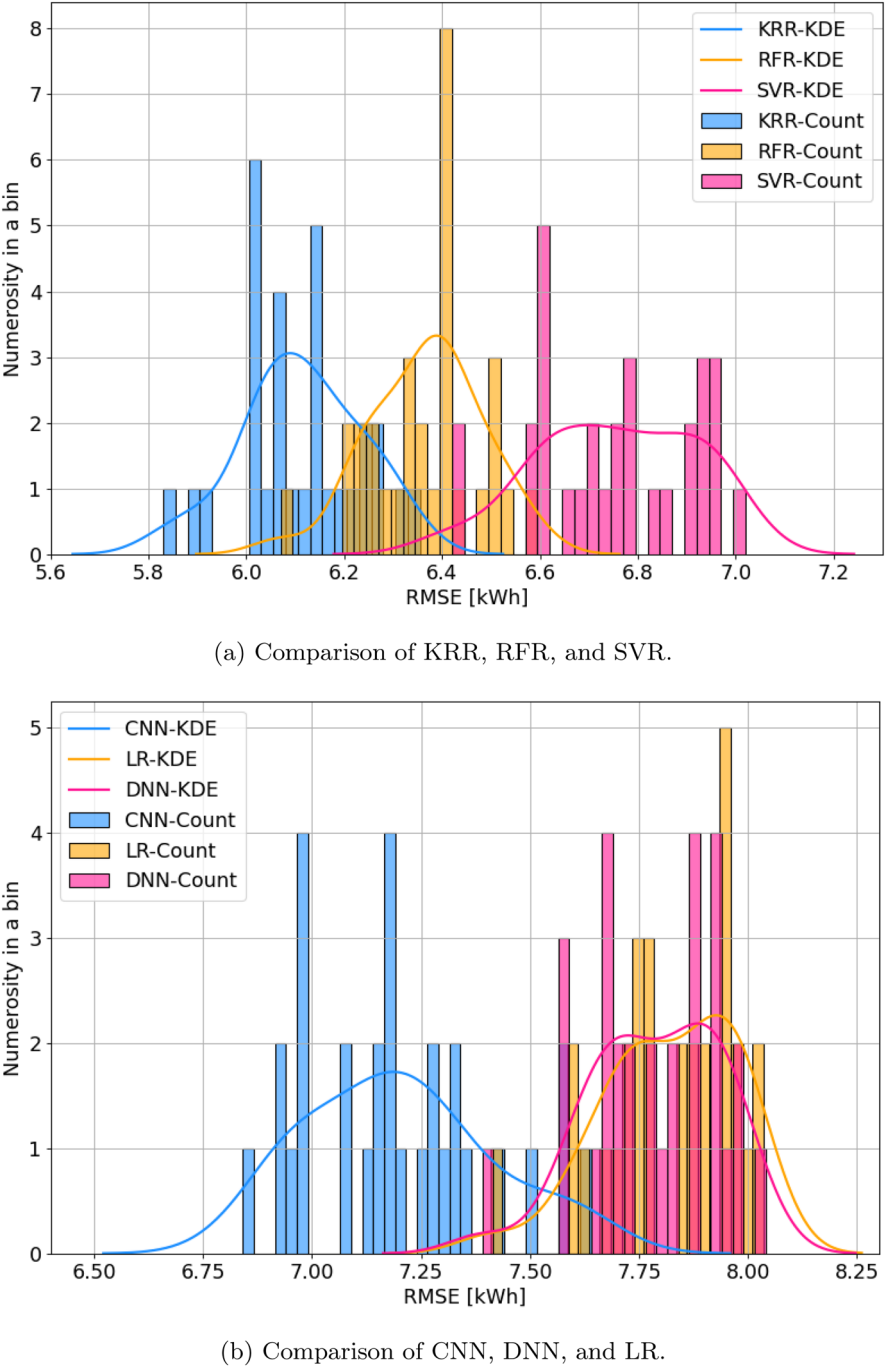
Comparison of the histograms and distributions of RMSE values. The models are trained and evaluated on the entire data

**Fig. 3:**
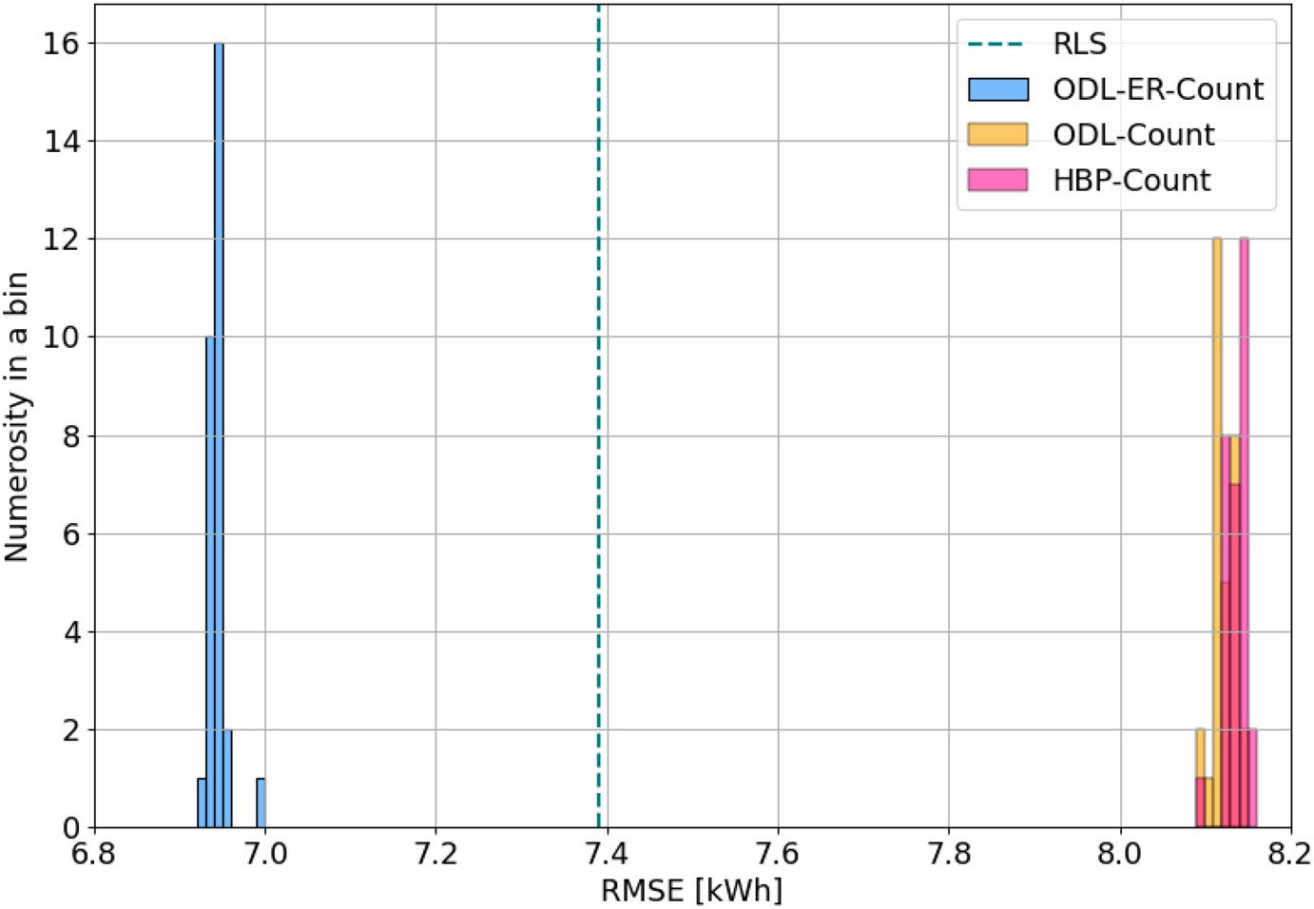
Comparison of the histogram and distribution of RMSE values for ODL-ER, RLS, ODL, and HBP (left to right). The models are trained and evaluated on the entire data. Note that RLS is deterministic (zero variance), forming a dirac delta

Figures 2a, 3 compare the distributions of RMSE values for models trained or evaluated on the entire data (using 30 randomly split train and test sets). Kernel density estimation (KDE) is a non-parametric method for estimating the probability density function of a data set using a kernel function (here, Gaussian kernel). With KDE, conclusions can be made about the underlying population based on a finite sample of data. It operates on a histogram where data points (in our case, the errors of individual runs) are assigned a bin, with ‘count’ referring to the number of RMSE values in a bin. In the supplementary information’s Sect. 6, the distribution of RMSE values is shown for all offline and online models.

Comparing the distributions of RMSE values for KRR, RFR, and SVR (cf. Fig. 2a) illustrates that KRR provides the best expected prediction, followed by RFR. KRR and RFR have a similar probability density function, exhibiting a lower dispersion of errors than SVR.

The comparison of the distribution of RMSE values for LR, DNN, and CNN (cf. Fig. 2b) shows that the distributions of error values for the LR and DNN are very similar, which corroborates the result of the significance test, indicating that differences between the two models might be based on a statistical coincidence. Moreover, the comparison shows that CNN makes the best prediction of the ANNs. The RMSE values of the CNN have a wide spread, indicating convergence into different local minima.

Except for the LSTM, the offline methods all perform similarly for forecasting the heat demand of the summer months (about 3.53 kWh to 4.4 kWh). The demand is difficult to forecast because no clear relations, patterns, and trends are shown in the data due to the composition of heat demand in the summer months. It is plausible that the selected input features do not sufficiently capture the motivation of users to use hot water.

ANNs, particularly LSTMs, are unsuitable for limited data, as already noted. LR and DNN do not model the demand peaks. As discussed above, the prediction of peak demand is particularly critical. The prediction error of the computationally intensive DNN hardly differs from the computationally efficient LR. The CNN has the best forecast quality among the ANNs, but it does not come close to the performance of the KRR or RFR.

KRR and RFR models are most suitable for practical application due to the low RMSE and MAD values. The advantage of RFR over KRR is that it is less data dependent, as indicated by the lower MAD value (different splits lead to more evenly generalizing models). However, KRR is more computationally efficient than RFR and far easier to understand and interpret for users with a limited data science background. This is especially apparent in comparison to all tested ANNs. Which of the two candidate models is more suitable must be determined on a project-specific basis. If computational and time requirements are generous, RFR can be used, otherwise, KRR is to be preferred. For our specific use case, the RFR is suitable because the controller has sufficient calculation power, and the calculations of the predictions are made only once a day.

### Online machine learning

As the data arrives as a stream, online ML models require—often significantly—less storage for training than offline ML models, even if the model has the same number of parameters. This can overcome memory problems in embedded systems used in real-world scenarios. They allow for quick model updates and adapt better to changes in the data.

To avoid long convergence times, online models require good initialization. This is problematic because validation data is not available in reality. Frequent model updates could disrupt convergence, as observed for SGD regression. Furthermore, the models are usually more challenging to maintain. Contaminated data can destabilize and corrupt models. Therefore, the models’ data and performance must be continuously monitored to avoid this. Maintenance is a critical issue when online ML models are used at the customer’s site. It must be guaranteed that the incoming data is error-free and not contaminated so that the online model is not corrupted and provides stable forecasts.

The online ML methods examined are discussed in more detail below.

#### Stochastic gradient descent

In an online learning environment, SGD is computationally very fast as few data points are processed. However, due to the frequent updates, the gradient descent towards minima is noisy, often leading in other directions and disrupting convergence. This explains the high error values and is indicated by the high MAD and RMSE values. SGD is unsuitable for practical application.

#### Recursive least squares

RLS is simple to calculate, mathematically understandable, and easy to implement. It has good convergence properties. Since the RLS is an adaptive filter algorithm, its prediction does vary over different runs. RLS is computationally light but potentially unstable, although the results do not confirm this. Only the forgetting factor, which is close to one, and the initialization value between zero and one need to be optimized. Due to these points, it is also possible for people with limited ML expertise to quickly learn to configure and apply RLS. RLS can be used directly on the controllers as it does not require large computing and storage capacities.

#### Online deep learning

The advantage of ODL is that it is the intuitive implementation of a DNN in an online learning environment. The performance of a DNN is highly dependent on parametrization, which is difficult to optimize in an online environment without substantial prior expert knowledge, which is hard to transfer to the resolution of this complex data science task, even given the obvious availability of civil engineering expertise. In this study, we optimized ODL for this specific use case, which is impossible in real-world scenarios due to a lack of validation data. Consequently, the prediction quality of ODL can be significantly worse in other projects where parametrization is not possible in advance. As with offline ML methods, training on CPUs is computationally costly and time-consuming. It is recommended to perform the calculation on an external GPU.

#### Online deep learning with experience replay

ER tries to overcome the limitations of ODL, at least partially. Previous experience is used efficiently by including it several times in the learning phase. In this way, ER addresses the problem of catastrophic forgetting. Furthermore, it has a better convergence behavior during training because the inputs are independent and identically distributed. In a way, it can be thought of as interacting with data like an offline method would. These changes result in the RMSE value for the samples being smaller than that of ODL. ER inherits the problems of parametrization from ODL. In addition, computation time and memory usage increase with the size of the buffer. Storing the experience in the buffer negates the advantage of online learning that less memory is required. The increasing memory requirements and high computational effort mean that ER cannot be trained directly on the embedded system.

#### Hedge backpropagation

HBP solves the problem of model architecture faced by ODL and ER by design. Due to the adaptive complexity, no fixed depth and width of the DNN has to be determined in advance. A disadvantage of the HBP architecture is that the adaptive capacity and the weighted predictions may not fully exploit the potential of a DNN. It requires additional parameter optimization and cannot be trained on-chip. Additionally, as all neural network–based methods, it suffers from poor explainability of predictions.

#### Statistical analysis

Using frequentist statistical testing, the null hypothesis is rejected for all RMSE values of the different models except for ODL and HBP, which are trained and evaluated with the winter months data. Therefore, the quantities of the RMSE values or the forecast quality of all models differ significantly and are probably not based on a statistical coincidence. However, as those tests can offer misleading results, we further investigate the distributions of the results in the following.

Figure 3 compares the distribution of RMSE values for RLS, ODL, ODL-ER, and HBP (excluding SGD as it was clearly not competitive). It is clear that ODL-ER shows the lowest prediction errors for the online ML models and that statistical coincidences are highly unlikely. Note that no probability density function is calculated for the RLS since its RMSE values have no variance. The distributions of RMSE values for ODL and HBP are very close and, while formed differently, it is unclear if differences (and advantages) are practically significant.

The models do not predict peak load demand in winter, a critical limitation for practical application. The naive model for the calculation of the MASE shows that, on average, the heat demand 1 h before is a better forecast than the prediction made by the model, especially in the winter months. This is primarily related to the poor peak load predictions but discourages real-world application of those models.

Different models are recommended for different application scenarios. Given its low error values and forecast quality, ODL-ER is preferred if the forecasts can be calculated externally and data for parametrization is available. Due to its adaptive model capacity, HBP should be used when validation data is unavailable. For this model, the calculation must be carried out externally. RLS can be used if the calculation has to be performed directly on the controllers.

In real-world scenarios, the online ML methods are the most suitable models for projects with insufficient data due to their adaptability. They can learn unknown situations and grasp concept drifts. If the data situation is sufficient, an online ML model can be replaced in the project process by an offline model, which provides a better prediction quality.

## Prediction of anomalous heat consumption

In addition to the main experiment, we investigated how well the offline and online ML methods can predict anomalies in consumption. An anomaly is a data point significantly different from the other observations. Predicting anomalous data, such as sudden cold snaps, is essential for a critical task such as predicting heating demand, as an adequate heat supply to the occupants must be guaranteed and not interrupted due to an incorrect model prediction. This could, in turn, also lead to legal consequences for the heating system operator. A model cannot make reliable predictions if it cannot deal with anomalous consumption. One possible scenario would be for a model to fail to respond to a cold snap. The heat consumption increases dramatically, and the model predicts too low heat consumption so that the heat supply to the residents can no longer be guaranteed.

### Detection of anomalies

The anomalies are detected using the isolation forest algorithm (IF) [73]. IF can detect anomalies in a multidimensional space. The idea of IF is that anomalous instances in a data set can be more easily separated or isolated from the rest of the samples than normal instances. Anomalies occur less frequently than normal data points and are further away from regular observations in the feature space.

The IF identifies 1597 anomalies, which corresponds to a percentage of 10.49 %. Of the anomalies, 320 (20.04 %) are in the summer, and 1277 (79.96 %) are in the winter months.

### Results

The RMSE for anomalies (cf. Table 9) is comparable to the RMSE values for all data points. KRR has the lowest values, and LSTM has the highest.

**Table 9:** Comparison of the RMSE values of the anomalies in kWh of the seven offline ML methods for the different periods summer, winter, and all year

Offline machine learning - Anomaly (RMSE [kWh])							
	LR	KRR	SVR	RFR	DNN	CNN	LSTM
Summer	4.02	**3**.**52**	3.67	3.55	3.91	3.83	5.45
Winter	9.26	**7**.**19**	8.05	7.59	9.21	8.44	16.07
All	7.86	**6**.**15**	6.83	6.53	7.81	7.26	17.82
All-summer	4.26	**3**.**4**	3.59	3.59	4.28	8.38	15.38
All-winter	9.53	**7**.**36**	8.3	7.53	9.45	8.87	18.97

Unlike the offline ML models, the RMSE values of online ML models are higher for anomalies than for the entire data set (cf. Table 10). Again, ODL-ER is among the online ML models and has the lowest RMSE values for the anomalies. However, the RMSE values are higher than for the DNN or CNN of offline ML models. RLS has lower RMSE values for anomalies than ODL or HBP, which have comparable values. As for general prediction, SGD is not performing competitively.

**Table 10:** Comparison of the RMSE values in kWh for the anomalies of the five online ML methods for the forecast horizons of 24 h and for the different periods summer, winter, and all year

Online machine learning - Anomaly (RMSE [kWh])					
	RLS	SGD	ODL	ODL-ER	HBP
Summer	6.27	9.62	8	**5**.**25**	7.21
Winter	12.18	40.44	15.71	**9**.**95**	16.57
All	11.58	33.43	14.49	**9**.**53**	14.73
All-summer	5.88	9.71	7.58	**5**.**34**	6.46
All-winter	12.55	36.86	15.68	**10**.**26**	16.07

### Discussion

All investigated offline ML methods cope well with anomalies. RMSEs for anomalies of the models are in the same order of magnitude as for the whole sample. KRR is robust against anomalies due to its L2 regularization preventing overfitting. In addition, RMSE values of the anomalies for the three ANNs are very high, indicating too low generalization.

For the online ML models, it is worthwhile to look at the time course of the prediction error for further investigation of the anomalies. For the time course of the RMSE values for the five online machine learning models trained and evaluated on the whole data set, see supplementary information Sect. 7.

All models have a very similar course of RMSE values; only the magnitude differs. The plots show that the RMSE values for the second winter are higher than for the first winter. There is little difference in the magnitude of the RMSE values between the first and second summer. Throughout the year, there are always smaller and larger peaks of the RMSE values that are also in the same time frames for all models. The peak around 30. November 2021 is particularly noticeable. During this period, all models have a problem making an accurate forecast. All data points from 29. November 2021, 21:00 to 01. December 2021 20:00 have been classified as anomalies. Therefore, this period is examined in more detail to determine how the individual online ML models react to and deal with anomalies.

**Fig. 4:**
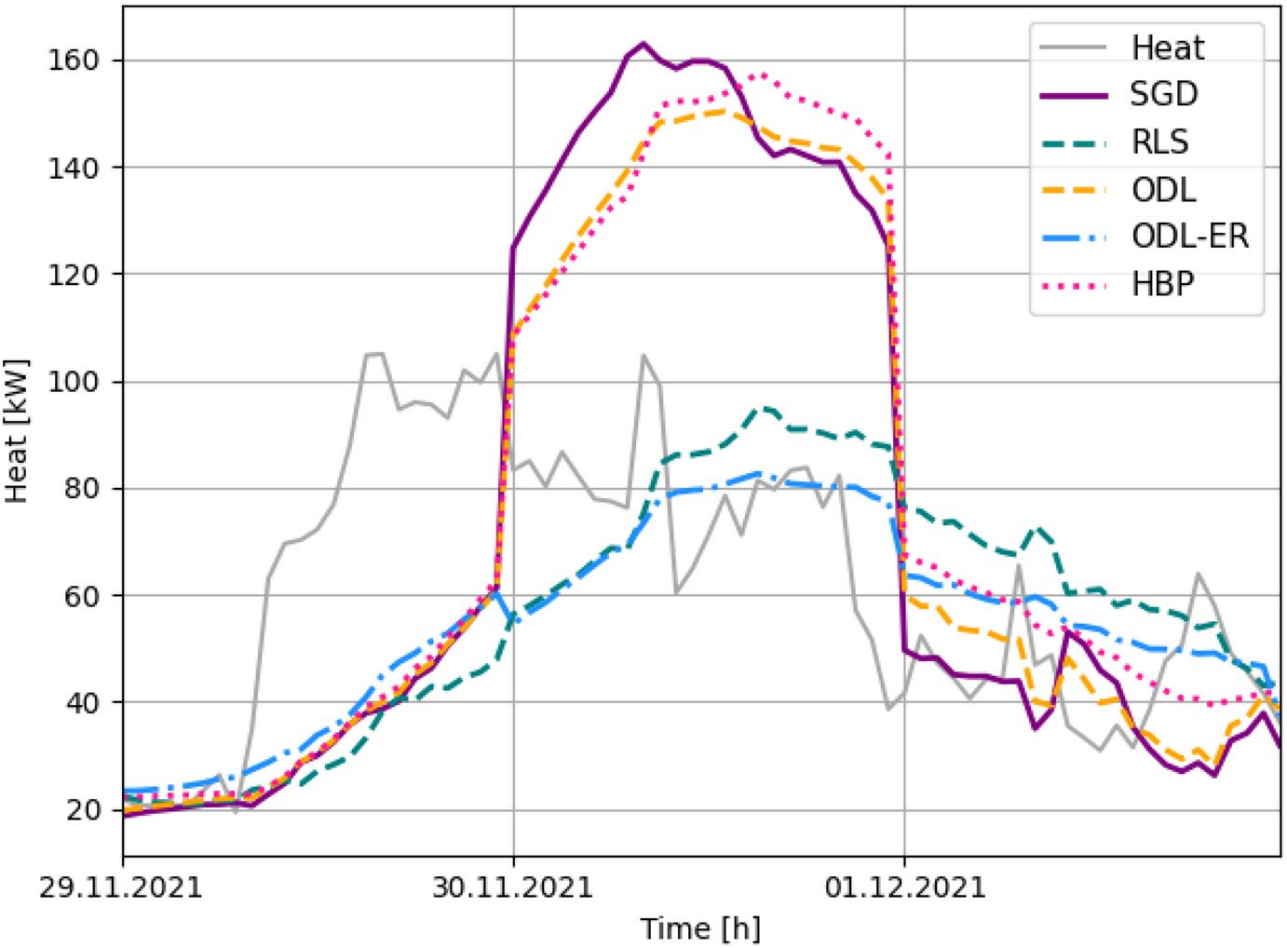
Comparison of the prediction of the five online machine learning models with the measured heat consumption from 29. November 2021 to 01. December 2021

As can be seen in Fig. 4, there is a sharp increase in heat consumption around 08:00 on 29. November, 2021. The high heat consumption persists throughout the day and slowly decreases over the next two days. The forecast for 29. November is still relatively similar for all models. Only when the models are updated with the data of the day with the abrupt increase in heat demand, the predictions for the next day differ, sometimes significantly. The SGD, ODL, and HBP are overcompensating for the new situation and making far too high predictions for 30. November 2021. RLS and ODL-ER are slow to adapt to the new situation. Their error values are lower than those of the SGD, ODL, or HBP, although they overcompensate later, and if heat demand had risen or stayed similarly high, they might have reacted too little or at least too late. After another update, the predictions of the models for the 01. December 2021 converge again.

## Conclusion and outlook

The paper investigated simple time series analysis methods and offline and online machine learning (ML) methods for predicting the heat demand of residential units in a specific scenario for which hardware specifications and also first data were available. The possibility of application in this real-world use case influenced the selection of methods. The new quarter we selected as a case study is comparatively small with only 66 units connected to the centralized heating system, and data has been available for 1.75 years. The results of this study will soon be used in the field in the form of a cloud application with distributed edge devices to enable remote training.

We found that, for this use case, a comparably high deviation of forecast and consumption in the summer months, where heat demand is fully based on hot water usage, is unavoidable with the available features due to erratic usage patterns among the limited number of occupants. The methods cannot model domestic hot water demand well and are therefore not perfectly suited for summer. Although, we want to stress that they do outperform a naive model. If the number of residents was higher, individual deviations in consumption patterns would not affect the hot water demand as much, making the methods more suitable. However, the additional space heating demand in the winter months is predictable as it is dependent on features such as daytime and outside weather. Interestingly, models trained and evaluated only on one seasonal subset do not outperform models trained on all data but evaluated on the same subset.

Additionally, we investigated whether there is a significant difference between a 24 and a 48 h forecasting window, finding that all methods performed comparably on the longer forecasting window.

In general, we investigated three families of methods:

Time Series models (Holt-Winter Smoothing and Seasonal ARIMA) were unable to convincingly perform on the data available and did not even beat a naive model, which takes as a prediction the heat consumption of 24 h ago.

Offline ML models performed the best out of the three. Interestingly, the more complex deep learning methods were outperformed by much simpler kernel-based (kernel ridge regression (KRR) and support vector regression) and ensemble-based (random forest regression) approaches. This might be due to the limited data availability or simply because underlying patterns between available features and heat demand are not so complex that they would warrant those methods. Given the remaining prediction errors, we can, however, suspect that the features that were gathered over the selected time period are not sufficient to describe all usage. Based on civil engineering knowledge, we are unaware of specific features that might have improved this other than more weather data and better forecasting thereof.

The kernel-based approaches are not only easier to implement but also to understand for engineers. An easier implementation allows engineers without a strong computer science background to debug and maintain the code base while still getting useful forecasts in return. Moreover, such an interpretable/comprehensible approach allows us to improve the energy management systems (its overall installation) rather than only the prediction system and, overall, gain a better understanding of the relationships between feature characteristics and heat demand needs. The great disadvantage of offline ML models is that they need large amounts of data before they can be first deployed and can, therefore, not go online with the energy management system itself. They are also vulnerable to concept and sensor drifts as they do not prioritize newer information over old data.

Theoretically, this is where online ML models should shine. However, in our data, drifts (besides the obvious seasonalities) seem to not have occurred yet. In general, the online methods did not perform competitively to their offline counterparts in terms of prediction errors and the critical peak demand forecasting. In a more detailed analysis, we found that all of the methods do lack behind in their forecast, while some overcompensate heavily. The best performing methods were the simple recursive least squares (RLS) algorithm and a deep learning approach where we used an experience replay–like buffer to sample mini-batches from for training whenever a new data point became available to the model.

In addition, it should be noted that the data was gathered during the COVID-19 pandemic period. To contain the pandemic, various measures were enacted in Germany, causing people to spend more time at home (e.g., increased work from home, lockdowns, or closed restaurants, bars, and clubs). These non-static restrictions had obvious impacts on the consumption patterns. Therefore, future examinations into how consumption patterns have changed after the enactment of restrictions and whether the models can cope with such strong concept drifts can be important. Likewise, it should be critically investigated to what extent our data can be useful in a post-pandemic world.

Another extreme situation, but not yet represented in the data, is the Russian invasion of Ukraine, which started on 24. February, 2022. Undoubtedly, this will strongly impact usage patterns (and energy supply) in Germany, with many users likely keeping the heating below previous thermal comfort levels to save energy as well as money. Again, the resulting consumption patterns and models need to be re-examined.

Besides the already investigated methods, a wide variety of other approaches is available with many having been explored in other settings in the existing literature. For this specific case study, the effect of other environmental parameters, such as wind speed, cloud cover, or humidity, on the heat demand should be evaluated. A re-examination of time series analysis techniques could be considered when data representing long-term seasonal patterns becomes available in a few years.

Also, the forecast accuracy of the models for a 48 and 72-h period should be analyzed again in more detail. However, our initial findings are that the forecasting horizon does not play a major role. For a safe application in the real world, a 24-h forecast might be too short-term as power plants might exhibit substantially longer lead times. Even if it is just about energy storage needs, e.g., in pumped-storage hydroelectricity plants rather than hydrogen gas or nuclear power plants.

Models that operate according to the divide-and-conquer principle in offline ML can be further investigated. The problem space can be divided into smaller spaces for which individual models, called experts, are trained. Another model or function can be used as a transition. This requires a more detailed data analysis to partition the problem space appropriately. One approach is Mixture of Experts (MoE), which divides the problem space between a few experts monitored by a gating network. Typically, these experts are individual smaller neural networks. This could allow for the more obvious expert models for summer, winter, and transition periods but also for models that are good at forecasting in certain weather settings or after certain usage patterns.

Additional methods can also be evaluated for online ML, e.g., XCSF [74], a highly interpretable rule set learning algorithm, or Online Random Forests [75], an incremental version of the Extreme Random Forest. Another promising approach is an online error correction presented in [76]. ANNs are used for prediction, which is combined with error correction methods.

The immediate next step will be to test selected methods in online applications, for example, starting with RLS, which is then later replaced by KRR. With these models, no data science experts are required for commissioning and maintenance.

Overall, we conclude that in this and similar settings online ML methods should only be used in the beginning and soon be replaced by offline ML approaches. However, engineers should keep the models in semi-regular observation in case of drifts (or directly deploy mechanisms to detect those). In a cloud-based training scenario, the algorithmic complexity is less important, as long as the models can run on the non-allocated hardware. Whether explainability is important needs to be determined on a case-by-case basis, but explainable methods are available and competitive.

## Supplementary information

The supplementary information for this paper is available at https://github.com/NeeleKemper/residential-unit-heat-forecast/tree/main/supplementary_information.

## Data Availability

The data used in this paper is available at https://github.com/NeeleKemper/residential-unit-heat-forecast/tree/main/src/data_processing/data. The data has already been cleaned and prepared for the study.

## Footnotes

1 https://github.com/NeeleKemper/residential-unit-heat-forecast/tree/main/supplementary_information.

2 For quarters where the heating system turns on/off at certain dates of the year, whether that date has passed should be included in the features (or different models should be trained altogether). However, in our case, study, the heating system was available year-round.

3 https://www.weatherbit.io.

4 https://www.phytec.de/produkte/single-board-computer/phyboard-regor-am335x/.

## Code availability

The code developed for this paper is available at https://github.com/NeeleKemper/residential-unit-heat-forecast/tree/main/src.
